# Herbivore assemblages affect soil microbial communities by altering root biomass and available nutrients in an alpine meadow

**DOI:** 10.3389/fpls.2023.1117372

**Published:** 2023-03-02

**Authors:** Yuzhen Liu, Xinquan Zhao, Wenting Liu, Xiaoxia Yang, Bin Feng, Chunping Zhang, Yang Yu, Quan Cao, Shengnan Sun, A. Allan Degen, Zhanhuan Shang, Quanmin Dong

**Affiliations:** ^1^ Academy of Animal Science and Veterinary Medicine, Qinghai University, Xining, Qinghai, China; ^2^ Qinghai Provincial Key Laboratory of Adaptive Management on Alpine Grassland, Qinghai University, Xining, Qinghai, China; ^3^ State Key Laboratory of Plateau Ecology and Agriculture, Qinghai University, Xining, Qinghai, China; ^4^ College of Animal Science and Technology, Yangzhou University, Yangzhou, Jiangsu, China; ^5^ Desert Animal Adaptations and Husbandry, Wyler Department of Dryland Agriculture, Blaustein Institutes for Desert Research, Ben-Gurion University of the Negev, Beer Sheva, Israel; ^6^ State Key Laboratory of Herbage Improvement and Grassland Agro-ecosystems, College of Ecology, Lanzhou University, Lanzhou, China

**Keywords:** herbivore assemblage, soil microbial community, plant community, Qinghai-Tibetan Plateau, β-diversity of soil microbes

## Abstract

Three different herbivore grazing assemblages, namely, yak grazing (YG), Tibetan sheep grazing (SG) and yak and Tibetan sheep co-grazing (MG), are practiced in alpine meadows on the Qinghai-Tibetan Plateau (QTP), but the effects of the different herbivore assemblages on soil microbes are relatively unknown. The microbial community plays an important role in the functional stability of alpine grassland ecosystems. Therefore, it is important to understand how the microbial community structure of grassland ecosystems changes under different herbivore grazing assemblages to ensure their sustainable development. To fill this gap, a field study was carried out to investigate the effects of YG, SG, and MG on plant communities, soil physico-chemical properties and microbial communities under moderate grazing intensity in alpine meadows. Grazing increased the β-diversity of the bacteria community and decreased the β-diversity of the fungal community. The herbivore assemblage affected the microbial community diversity, but not the plant community diversity. Total phosphorus, soil bulk density, root biomass, and plant α-diversity were correlated with both the bacterial and fungal community composition, available phosphorus and soil moisture were correlated only with the bacterial community composition, while available potassium and above-ground net primary production (ANPP) were correlated only with the fungal community composition. Soil available nitrogen, soil available phosphorus and soil bulk density were highest in SG, while ANPP was highest in MG. It was concluded that MG can improve ANPP and stabilize the soil microbial community, suggesting that MG is an effective method for sustainable use and conservation of alpine meadows on the QTP.

## 1 Introduction

Grazing can alter the grassland ecosystems by affecting the soil physico-chemical properties (Liu et al., 2015; Jordon, 2021). For example, livestock trampling compacts soil to increase soil bulk density and alters soil water-holding capacity (Veldhuis et al., 2014; Byrnes et al., 2018). In addition, faeces and urine from herbivores increase soil nitrogen (N) availability and reduce soil pH (Zhou et al., 2017; Hong et al., 2021).

The changes in soil physico-chemical properties caused by grazing, trampling and faecal and urine deposition directly or indirectly affect the microbial community, which in turn affects grassland ecosystem function (Yang et al., 2013; Liu et al., 2015; Zhang et al., 2020) (**Figure S1**). In addition, grazing, especially selective grazing by different herbivores (Pan et al., 2019), not only alters the plant community composition and decreases the above-ground biomass, but also increases the below-ground biomass. This promotes an increase in plant root exudates, which include organic substances, polysaccharides and enzymes (Bardgett et al., 1998; Huhe et al., 2017; Gao et al., 2021). These root exudates influence microbial abundance, such as increasing the abundance of pathogenic bacteria, and also drive plant-soil-microbes feedbacks in response to plant needs, where plants can adjust their exudation patterns to tailor microbial recruitment to meet nutrient requirements (Zhao et al., 2021).

The alpine meadows of the Qinghai-Tibetan Plateau (QTP), one of the largest natural grasslands in China, cover an area of approximately 2.27 × 10^6^ hm^2^, and provide important ecological functions, as well as social services for the inhabitants (Cao et al., 2021). The QTP is also a huge carbon (C) sink, with a total organic C stock of approximately 19.23 Pg C, accounting for 23% of China’s total C pool (Yang et al., 2017). Primary production on the plateau is generally low due to the unfavorable climate and short growing season, and the ecosystem is highly sensitive to external disturbances (Lu et al., 2017). In recent decades, the grasslands have been significantly degraded due to overgrazing, irrational management and global climate change (Harris, 2010; Wu et al., 2014; Xue et al., 2017). Poor grassland management has reduced plant diversity and soil fertility and lessened ecosystem function (Cao et al., 2011; Niu et al., 2019).

Grazing is the most common land use in alpine meadows and crucial for the livelihoods of local people, and is, therefore, essential for maintaining the biodiversity and ecosystem function of alpine meadows (Niu et al., 2016; Lu et al., 2017; Wang et al., 2019). Current research on alpine meadows has focused on the effects of grazing exclusion, grazing intensity and practices (rotational or deferred grazing) on plant community composition, plant productivity, soil respiration and microbes (Chen et al., 2016; Du et al., 2020; Jäschke et al., 2020).

To prevent further degradation of grasslands, the Chinese government has enacted a series of grassland use and protection policies, such as extensive fencing to exclude grazing (Sun et al., 2020). In addition, the government divided the grasslands so that each herder has private pasture for livestock. Some herders graze only Tibetan sheep or yaks, while others co-graze yaks and Tibetan sheep. The herbivore assemblage affects the soil biotic and abiotic properties, which then influences the plant species, bacteria and fungi communities and the grassland ecosystem function (Eldridge et al., 2021; El Mujtar et al., 2021). The key role of microbes in maintaining the sustainability of grassland ecosystems has been widely recognized (Rillig et al., 2019). However, soil microbes have also been identified as a critical factor in the degradation of alpine meadows (Chen et al., 2021). Therefore, understanding how soil microbial communities respond to different herbivore assemblages could provide important information for the management of degraded grasslands. However, there are very few studies on the effects of herbivore assemblages on soil microbial communities. To fill this knowledge gap, we designed a field study to assess: (a) how different herbivore assemblages affect the soil microbial community and composition; and (b) what environmental factors interact with the herbivore assemblage to impact the soil microbial communities. We hypothesize that the herbivore assemblage influences the environmental factors (soil physico-chemical properties and the composition of the plant community). To test this hypothesis, we examined three different herbivore grazing assemblages, namely, yak grazing, Tibetan sheep grazing and yak and Tibetan sheep co-grazing, under moderate grazing intensities to investigate the effects on the soil physico-chemical properties, microbial communities and plant communities on the QTP.

## 2 Materials and methods

### 2.1 The study site

This study site was situated in the Xihai Town, Qinghai Province (lat. 36°44′-37°39′ N, long. 100°23′-101°20′ E; 3000 m), in eastern QTP (**Figure S2**). The climate type is mountain plateau, with an average annual precipitation of 330~370 mm, mainly concentrated in May to September (growing season). The annual average temperature is 1.4°C, with the coldest monthly average temperature of -24.8°C in the non-growing season, and the hottest monthly average temperature of 12.5°C in the growing season. The grassland type is alpine meadows nd the soil type is clay loam. The main vegetation was *Kobresia humilis*, *Elymus nutans*, *Stipa sareptana*, *Potentilla acaulis* and *Leymus secalinus* (Yang X. et al., 2019).

### 2.2 Design of experiments

Grazing plots with different herbivore assemblages were established in June 2014 in an alpine meadow with flat terrain and relatively uniform environment and plant cover. Twelve plots were arranged in a randomized design, with three plots each for yak grazing (YG, 2600 m^2^), Tibetan sheep grazing (SG, 1700 m^2^), yak and Tibetan sheep co-grazing as 1:2 mixed grazing (MG, 4300 m^2^), and grazing exclusion (NG, 500 m^2^) treatments. Grazing intensity was moderate at 0.5 sheep units/ha, as it was previously reported that above-ground biomass and plant α-diversity were greatest in alpine meadows under moderate grazing intensity (Gao and Carmel, 2020). The yaks (~ 100 kg and 1.5-year-old males) and the Tibetan sheep (~30 kg and 1 year old males) were purchased each year from local herders at the start of the grazing season and were de-wormed prior to the study. The livestock grazed during the growing season (June to October) each year for 7 years, till August 2020, and the forage the grazed plots was 50% - 55%. There was no supplementary feed offered during grazing, but drinking water was available freely.

### 2.3 Plant and soil sampling

In 2020, three cages (0.7m × 0.7m) spaced more than 10 m apart were randomly placed in each plot before grazing, and the biomass in a 0.25 m^2^ quadrat in each cage was collected to measure the above-ground net primary productivity (ANPP). At the end of August, all above-ground biomass was harvested within the quadrats in the 36 cages (4 treatments × 3 replicates × 3 cages), and was dried at 85°C to constant weight (Wang et al., 2014). The plant community characteristics was surveyed in three 0.25 m^2^ random quadrats per plot, and the abundance, height, richness and cover of each species were recorded (Wang et al., 2021). The above-ground parts of the plants were pruned, and were dried at 85°C (24 h) in an oven. The plant species were divided into four functional groups based on plant characteristics: Gramineae, Cyperaceae, Leguminosae and forbs, respectively. Concomitantly, we collected the root biomass in the 0-10 cm (Yang F. et al., 2019).

Surface soil samples (0-10 cm) for DNA extraction and sequencing were collected from the same quadrats used for surveying plant species. Three random soil cores (0-10 cm) were taken in each quadrat using a 7 cm soil auger. The samples from each quadrat were mixed thoroughly to form one composite sample for the determination of relevant indicators. Mixing multiple soil samples reduces errors due to soil heterogeneity (Edwards, 2010), and many studies on soil microbiology and phyisco-chemical properties often use such an approach (Chen et al., 2010; Huhe et al., 2017; Xiao et al., 2020). These 36 soil samples were stored at 4°C and immediately brought back to the laboratory.

### 2.4 Plant and soil measurements

The importance value (IV) was calculated according to the following formula (Zhang et al., 2016): *IV*=(*RH_i_* + *RC_i_*)/2, *RH* was relative height and *RC* was relative cover in a community. The composite soil sample was sieved through a 2 mm screen to remove impurities such as stones and debris. The roots were washed and dried at 85°C (48 h) to constant weight to measure root biomass. The soil was then divided into three sub-samples for the determination of different indicators. One sub-sample was air-dried at room temperature (25°C) and used to measure the soil physico-chemical characteristics, the second subsample was kept at 4°C for measurements of soil nitrate ( ${\text{NO}}_{3}^{-}$ ) and ammonium ( ${\text{NH}}_{4}^{+}$ ) contents, and the third subsample was kept at -80°C for DNA extraction.

We followed the method of Ye to determine the soil total nitrogen (TN), soil total carbon (TC) (Ye et al., 2018). The available phosphorus (AP), available potassium (AK), nitrate ( ${\text{NO}}_{3}^{-}$ -N), ammonium ( ${\text{NH}}_{4}^{+}$ -N) contents and soil pH were measured following Huang (Huang et al., 2019). The soil 0-10 cm bulk density (SBD) was measured by the cutting ring (100 cm^3^) method (Wang et al., 2021). ANPP calculation was based on the above-ground biomass in a quadrat (Zheng et al., 2022).

### 2.5 High throughput sequencing of soil samples

Five samples were selected randomly from each treatment for microbiological analysis. Sequencing was done by the Gene Denovo Biotechnology Co., Ltd (Guangzhou, China) (**Appendix S1**).

### 2.6 Data analyses

Plant community α-diversity was assessed using Shannon, Simpson and species richness indices, and soil microbial community α-diversity was assessed using Sobs, Chao1, Shannon and Simpson indices. One-way ANOVA was used to test for differences among herbivore assemblages for plant and soil physico-chemical variables and relative abundances of different taxa and α-diversity indices (*P*<0.05). Plant species and soil microbial β-diversity in herbivore assemblages were analyzed by nonmetric multidimensional scaling analysis (NMDS) (Cao et al., 2022). Based on canonical correlation analysis (CCA) using ‘enfit’, the main environmental factors driving changes in the microbial community were identified. Linear discriminant analysis effect size (LEfSe) was used to identify the main taxa driving changes in the microbial community under different treatments (Segata et al., 2011). We identified the most refined classification levels for bacteria (phylum level) and fungi (family level; **Figures 2**, **3**). A structural equation model (SEM) identified the relationships between soil bacterial and fungi β-diversity and environmental factors. All statistical analyses and drawings used R 4.0.2 for Windows and the “vegan”, “psych”, “piecewiseSEM” package.

## 3 Results

### 3.1 Effect of herbivore assemblage on plant traits and soil properties

${\text{NH}}_{4}^{+}$ -N, ${\text{NO}}_{3}^{-}$ -N and AP contents and SBD were highest in SG (*P*< 0.05), soil moisture was highest in YG (*P*< 0.05), root biomass and ANPP were highest in MG (*P*< 0.05), and AK content and shoot biomass were highest in NG. Herbivore assemblage had no effect on soil TN content, the C:N ratio and pH (**Table 1**).

**Table 1:** Effects of herbivore assemblage on biotic and abiotic properties.

Variables	YG	SG	MG	NG
TC (g·kg^-1^)	**40.0 ± 0.60b**	**42.8 ± 0.88a**	**40.9 ± 0.39ab**	**42.0 ± 0.79ab**
TN (g·kg^-1^)	3.22 ± 0.06	3.47 ± 0.08	3.24 ± 0.03	3.32 ± 0.10
C/N	12.4 ± 0.18	12.4 ± 0.31	12.6 ± 0.11	12.7 ± 0.28
TP (g·kg^-1^)	**0.155 ± 0.001ab**	**0.162 ± 0.001a**	**0.154 ± 0.003ab**	**0.153 ± 0.003b**
${\text{NO}}_{3}^{-}$ -N(mg·kg^-1^)	**9.70 ± 1.26b**	**12.81 ± 1.04a**	**3.66 ± 0.18c**	**3.57 ± 0.51c**
${\text{NH}}_{4}^{+}$ -N(mg·kg^-1^)	**13.4 ± 0.91b**	**16.7 ± 0.73a**	**6.55 ± 0.89c**	**1.50 ± 0.07d**
AP (mg·kg^-1^)	**3.78 ± 0.31c**	**4.59 ± 0.02a**	**4.31 ± 0.16b**	**3.52 ± 0.02d**
AK (mg·kg^-1^)	**83.9 ± 2.83c**	**89.4 ± 2.87c**	**116.3 ± 2.78b**	**131.3 ± 4.27a**
pH	7.98 ± 0.01	7.98 ± 0.02	7.98 ± 0.01	7.95 ± 0.03
Moisture (%)	**19.8 ± 1.08a**	**17.0 ± 0.30b**	**16.0 ± 0.86b**	**15.8 ± 0.11b**
SBD (g·cm^-3^)	**0.96 ± 0.01c**	**1.13 ± 0.01a**	**1.06 ± 0.01b**	**0.90 ± 0.02d**
Shoot biomass (g·m^-2^)	**168 ± 15.29b**	**170 ± 30.48b**	**135 ± 7.97b**	**230 ± 16.32a**
Root biomass (kg·m^-2^)	**1.76 ± 0.22b**	**1.80 ± 0.22b**	**2.52 ± 0.18a**	**1.59 ± 0.26b**
ANPP (g·m^-2^)	**230 ± 16.15b**	**221 ± 31.75b**	**286 ± 10.41a**	**230 ± 16.32b**

Means within a row with different lowercase letters (a, b, c, d) differ from each other (P< 0.05). Bold values represent significant differences among treatments. YG, Yak grazing; SG, Tibetan Sheep grazing; MG, Yak and Tibetan sheep mixed grazing; NG, No grazing. TC, soil total carbon; TN, soil total nitrogen; C/N, carbon/nitrogen; TP, soil total phosphorus; ${\text{NO}}_{3}^{-}$ -N, soil nitrate; ${\text{NH}}_{4}^{+}$ -N, soil ammonium; AP, soil available phosphorus; AK, soil available potassium; SBD, soil bulk density; ANPP, above-ground net primary productivity.

### 3.2 Effect of herbivore assemblage on plant and microbial community compositions

**Figure 1:**
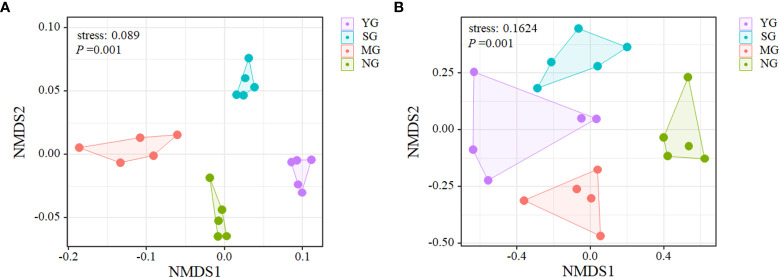
Non-metric multidimensional scaling (NMDS) ordination of all sampling units indicating the relative differences in bacterial **(A)** and fungal **(B)** community compositions. YG, Yak grazing; SG, Tibetan Sheep grazing; MG, Yak and Tibetan sheep mixed grazing; NG, No grazing.

There was no effect of herbivore assemblage on plant α- diversity (*P* > 0.05). Bacteria α- diversity was highest in SG (*P* > 0.05), and fungi α- diversity, and the sobs and Chao1 were higher in SG than NG (*P* > 0.05, **Table 2**). In addition, the Shannon and Simpson indices were higher in MG than SG (*P* > 0.05). NMDS analysis revealed that the bacteria and fungi communities differed among herbivore assemblages, but the plant communities did not (**Figure S3A**; **Figure 1**).

**Table 2:** α-diversity of plant, bacterial and fungal communities under different herbivore assemblages.

Groups	Diversity	YG	SG	MG	NG
Plant	Richness	14.6 ± 0.44	14.1 ± 0.74	13.6 ± 0.18	15.1 ± 0.74
	Shannon	2.23 ± 0.03	2.20 ± 0.06	1.93 ± 0.05	2.35 ± 0.05
	Simpson	0.77 ± 0.03	0.74 ± 0.04	0.73 ± 0.04	0.76 ± 0.03
Bacteria	Sobs	**3692 ± 37.8b**	**3847 ± 25.0a**	**3551 ± 42.5c**	**3619 ± 25.1bc**
	Chao1	**4453 ± 48.3b**	**4669 ± 27.9a**	**4221 ± 38.2c**	**4352 ± 35.2b**
	Shannon	**9.64 ± 0.01b**	**9.77 ± 0.17a**	**9.60 ± 0.01b**	**9.62 ± 0.02b**
	Simpson	**0.9967 ± 0.0002b**	**0.9970 ± 0.0006a**	**0.9966 ± 0.0005b**	**0.9967 ± 0.0004b**
Fungi	Sobs	**397 ± 38.8ab**	**419 ± 13.3a**	**367 ± 17.2ab**	**341 ± 14.6b**
	Chao1	**540 ± 45.0ab**	**552 ± 21.3a**	**485 ± 11.0ab**	**454 ± 23.1b**
	Shannon	**4.72 ± 0.24ab**	**4.11 ± 0.16b**	**4.96 ± 0.17a**	**4.43 ± 0.37ab**
	Simpson	**0.91 ± 0.24a**	**0.82 ± 0.03b**	**0.93 ± 0.01a**	**0.89 ± 0.03ab**

Means within a row with different lowercase letters (a, b, c, d) differ from each other (P< 0.05). Bold values represent significant differences among treatments. YG, Yak grazing; SG, Tibetan Sheep grazing; MG, Yak and Tibetan sheep mixed grazing; NG, No grazing.

**Figure 2:**
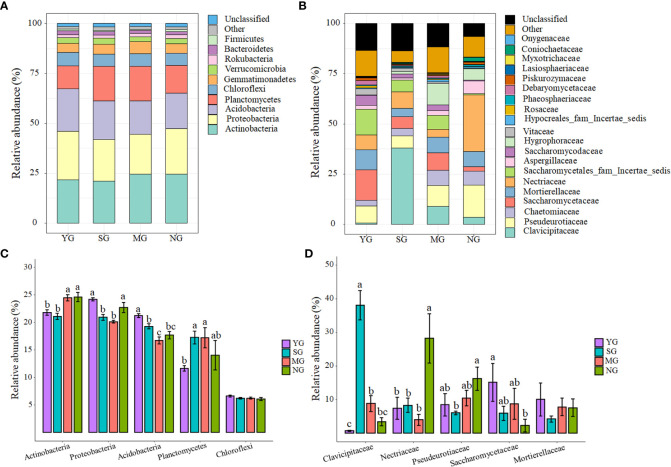
Relative abundances of bacterial phyla under different herbivore assemblages **(A)**; relative abundances of fungal families under different herbivore assemblages **(B)**; dominant bacterial phyla **(C)**; dominant fungal families **(D)**. Significance level: *P*< 0.05. YG, Yak grazing; SG, Tibetan Sheep grazing; MG, Yak and Tibetan sheep mixed grazing; NG, No grazing.

**Figure 3:**
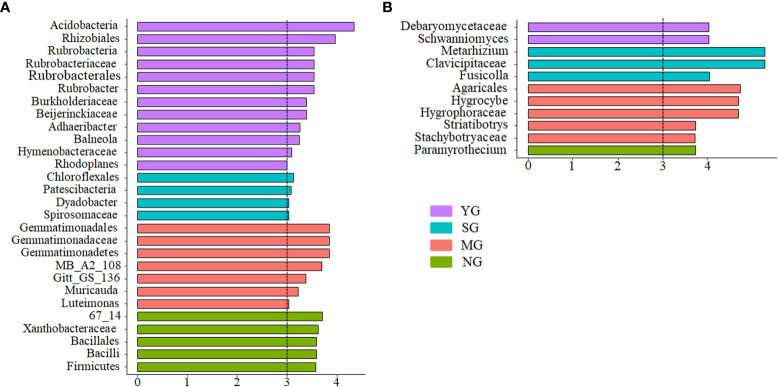
Indicator bacteria with LDA scores of 3 or greater in bacterial communities under different herbivore assemblages **(A)**; indicator fungi with LDA scores of 3 or greater in fungal communities under different herbivore assemblages **(B)**. YG, Yak grazing; SG, Tibetan Sheep grazing; MG, Yak and Tibetan sheep mixed grazing; NG, No grazing.

At the phylum level, Actinobacteria, Proteobacteria, Acidobacteria, Planctomycetes and Chloroflexi dominated the soil bacterial communities (**Figure 2A**). Only Chloroflexi did not differ among herbivore assemblages (**Figure 2C**). At the family level, Clavicipitaceae, Nectriaceae, Pseudeurotiaceae, Saccharomycetaceae and Mortierellaceae dominated the soil fungal communities (**Figure 2B**). Only Mortierellaceae did not differ among herbivore assemblages, while the relative abundance of Clavicipitaceae was higher in SG than NG (*P*< 0.05, **Figure 2D**). Further NMDS analyses of bacteria and fungi that differed significantly in relative abundances among herbivore assemblages revealed significant differences in the β-diversity of bacterial communities, but not in the fungal communities (**Figure S4**; **S5**). For all treatments, the phyla that caused the main changes in bacterial communities were Acidobacteria and Proteobacteria. In addition, the phylum that caused the main changes in fungal communities was Ascomycota (**Figure 3**).

### 3.3 Key factors driving changes in microbial communities

**Figure 4:**
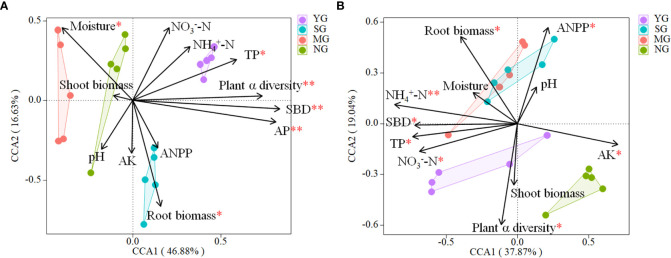
Canonical correspondence analysis using pooled data of bacterial **(A)** and fungal **(B)** communities and abiotic and biotic variables (arrows). The values of Axis 1 and 2 are percentages that the corresponding axis can explain. TC, soil total carbon; TN, soil total nitrogen; C/N, carbon/nitrogen; TP, soil total phosphorus; ${\text{NO}}_{3}^{-}$ -N, soil nitrate; ${\text{NH}}_{4}^{+}$ -N, soil ammonium; AP, soil available phosphorus; AK, soil available potassium; SBD, soil bulk density; ANPP, above-ground net primary productivity. YG, Yak grazing; SG, Tibetan Sheep grazing; MG, Yak and Tibetan sheep mixed grazing; NG, No grazing.

The CCA revealed that the bacterial community composition was correlated (P< 0.05) to soil TP, AP, moisture content, SBD, root biomass and plant α diversity (**Table S1**, **Figure 4A**). The fungal community composition was correlated (P< 0.05) to TP, ${\text{NO}}_{3}^{-}$ -N, ${\text{NH}}_{4}^{+}$ -N, AK, SBD, root biomass, ANPP and plant α diversity (**Table S1**, **Figure 4B**).

**Figure 5:**
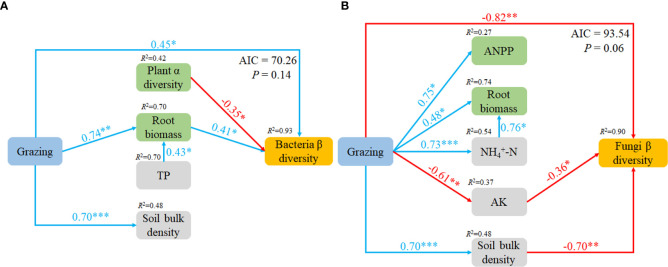
Piecewise structural equation model (SEM) describing the effects of grazing on soil bacteria **(A)** and fungi **(B)** β diversity. TP, soil total phosphorus; ${\text{NH}}_{4}^{+}$ -N, soil ammonium; AK, soil available potassium.

The variables which had significant effects on the microbial community compositions in CCA were selected as predictors to generate a piecewise SEM to identify the key drivers on soil microbial β-diversity. For bacteria, path analysis indicated that grazing, plant α diversity and root biomass had a direct impact on soil bacteria β-diversity, and grazing also increased root biomass and SBD. For fungi, the path analysis indicated that grazing, AK and SBD had direct impacts on soil fungi β-diversity, and grazing increased ANPP, root biomass, ${\text{NH}}_{4}^{+}$ -N and SBD, but decreased AK (**Figure 5**).

## 4 Discussion

### 4.1 Effect of herbivore assemblage on plant and soil properties

The herbivore assemblage affected soil available nutrients, but not total nutrients. Studies at the eastern edge of the QTP reported similar conclusions (Liu et al., 2022). Soil nitrate, ammonium and AP contents and SBD were highest in SG. A study in the eastern Eurasian steppe reported that soil available N was closely related to the herbivore assemblage, and that the highest available N content was with sheep grazing (Zhang et al., 2020). This was also observed in the current study. The faeces and urine of small herbivores have a relatively high N content and are easily degraded, while those of large herbivores have a relatively low N content and are not easily degraded (Wang et al., 2018). Consequently, this could explain why soil nitrate and ammonium nitrate contents were higher in grazed grassland than in NG grassland, and higher in SG than in YG and MG.

Tibetan sheep graze over a greater area and defaecate more frequently than yaks. Soil AP was highest in SG and soil AK was highest in NG. This may be related to the spatial pattern use of the different livestock (Yamada et al., 2011). Tibetan sheep require better quality forage than yaks (Laca et al., 2010), and, thus, are more selective in their intake. This difference forces sheep to walk over greater distances than yaks in search of high quality forage. The source of soil AP is mainly from mineralization, biofiltration and eluviation (Walker and Syers, 1976), while soil AK is mainly released from the soil parent material during soil formation. The AK is highly soluble and is easily lost due to external disturbances (Wang et al., 2002). The greater movement by Tibetan sheep than yak reduces plant cover, disrupts the soil crust and leads to increased mineralization and eluviation (Zhang et al., 2020). This could explain why the highest content of soil AP and lowest content of soil AK were recorded in SG. In addition, grazing reduced shoot biomass, but both below-ground biomass and ANPP were highest in MG. Grazing can drive plant biomass allocation to below-ground, and when subjected to external stresses, vegetation employs special defense mechanisms to allot more biomass to below-ground (Zhou et al., 2021). In MG, more of the plant species were subjected to foraging stress, as the Tibetan sheep and yak overlapped in many of the plant species consumed, but each also consumed some unique plant species so the biomass allocation to the below-ground was more pronounced. The highest ANPP occurred in MG, which indicates that the plants had a strong compensatory growth at this time.

### 4.2 Effect of herbivore assemblage on microbial α and β diversity

Grazing can affect soil microbial communities directly by altering resource availability, or indirectly through its effects on plants. In the current study, bacterial α-diversity differed among herbivore assemblages, with the highest in SG, but fungal α-diversity did not differ among herbivore assemblages. This suggests that the bacterial community was more sensitive to herbivore assemblages than the fungal community, which is contrary to the findings of Yang X. et al. (2019), whose research indicated that winter and annual grazing did not affect microbial α-diversity. These conflicting findings could be due to different herbivore assemblages, different grazing intensities and different initial microbial communities.

The β-diversity of both soil bacteria and fungi displayed differences among herbivore assemblages. Further analysis of the top 4 microbes in terms of relative abundances revealed that the β-diversity of bacteria differed among treatments, whereas the fungi did not. These results indicate that the difference in bacterial β-diversity was due to the dominant species, whereas in fungi was due to rare species. Theoretically, different plant characteristics and soil environments can create different niches (Hamid et al., 2021), and, consequently, affect specific soil microbial communities (Pot et al., 2022). In the current study, bacterial β-diversity was driven mainly by plant α-diversity and root biomass, but fungal β-diversity was driven mainly by AK and SBD. Some of the differences could be due to the selective intake by the herbivores and excrement which can affect plant and soil properties and can cause the different responses of microbial communities to grazing. The different responses of microbial communities to the presence of herbivores warrant further investigation.

### 4.3 Effect of herbivore assemblage on microbial community composition

Herbivore assemblage had a greater effect on soil microbial β-diversity than soil microbial α-diversity, indicating that microbial β-diversity is more sensitive to herbivore assemblage than microbial α-diversity. Another study on the effects of grazing on alpine grasslands came to similar conclusions (Cao et al., 2022). Soil nutrient contents, soil moisture content, SBD, root biomass and plant α-diversity were the main drivers of soil microbial community composition for herbivore assemblages, which is consistent with several studies under different grazing intensities (Yang F. et al., 2019; Cao et al., 2021; Wang et al., 2021) and may be due to: (1) soil microbes are sensitive to soil nutrient and moisture availability, particularly due to nutrient changes caused by urine and manure from grazing livestock, resulting in a shift in soil microbial communities from one dominant species to another (Levy-Booth et al., 2016); and (2) feedback loops between plant communities, soil and microbial communities change markedly under different herbivore assemblages (Rigg et al., 2016). Furthermore, litter deposition and changes in SBD by livestock affect soil permeability, which in turn affects the composition of soil microbial communities (Che et al., 2020).

## 5 Conclusions

Different herbivore assemblages directly or indirectly influence changes in microbial community structure through their selective foraging, trampling and excreta return pathways, which in turn affect the stability of grassland ecosystem structure and function. The results of this study show that grazing increased bacterial community β-diversity and decreased fungal community β-diversity. Soil available nitrogen (AN), available phosphorus (AP) and soil bulk density (SBD) were highest in SG, while ANPP was highest in MG. YG and SG reduced the relative abundance of Actinobacteria among bacteria, while SG increased the relative abundance of Clavicipitaceae among fungi. From a management perspective, the results of this study indicate that MG can improve ANPP and is less damaging to the soil microbial community and its functions than SG and YG. Therefore, co-grazing Tibetan sheep and yaks should be considered as a viable option for sustainable development of alpine meadows on the QTP.

## Data availability statement

The original contributions presented in the study are included in the article/**Supplementary Material**. Further inquiries can be directed to the corresponding author.

## Author contributions

QD and WL conceived the ideas and designed the study; YL, BF, QC, SS, CZ, XY, and YY collected the data; YL and XZ analyzed the data; YL wrote and revised the draft; and AD and ZS revised the manuscript. All authors contributed to the article and approved the submitted version.
