# Supplementary material for: Herbivore assemblages affect soil microbial communities by altering root biomass and available nutrients in an alpine meadow
Source: Front Plant Sci. 2023 Mar 2;14:1117372. doi: 10.3389/fpls.2023.1117372 (PMC10017739; doi:10.3389/fpls.2023.1117372)
Supplement: Supplementary file 1 [file DataSheet_1.docx]

Supplementary Material

**The effect of herbivore assemblage on soil bacterial and fungal communities in alpine meadow**

Yuzhen Liu, Xinquan Zhao, Wentin Liu, Xiaoxia Yang, Bin Feng, Chunping Zhang, Yang Yu, Quan Cao, Shengnan Sun, A Allan Degen, Zhanhuan Shang, Quanmin Dong^*^

*** Correspondence:** Quanmin Dong, E-mail: [qmdong@qhu.edu.cn](mailto:qmdong@qhu.edu.cn)

# Supplementary Figures and Tables

## Supplementary Figures


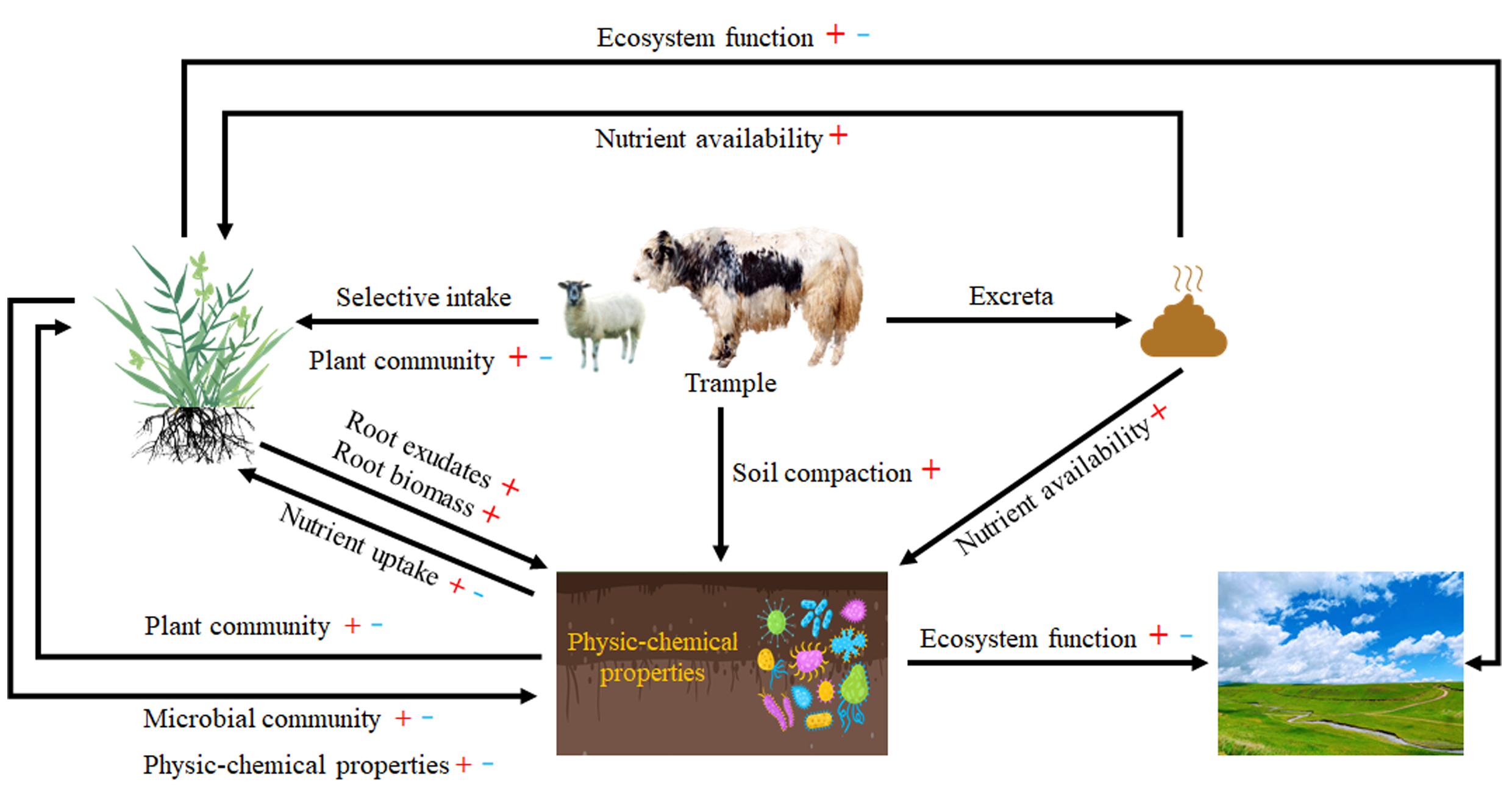


**Supplementary Figure 1.** Conceptual illustration showing the potential links among grazing, plant and soil microbial communities, and ecosystem function. “+” represents positive effects, and “-” represents negative effects.


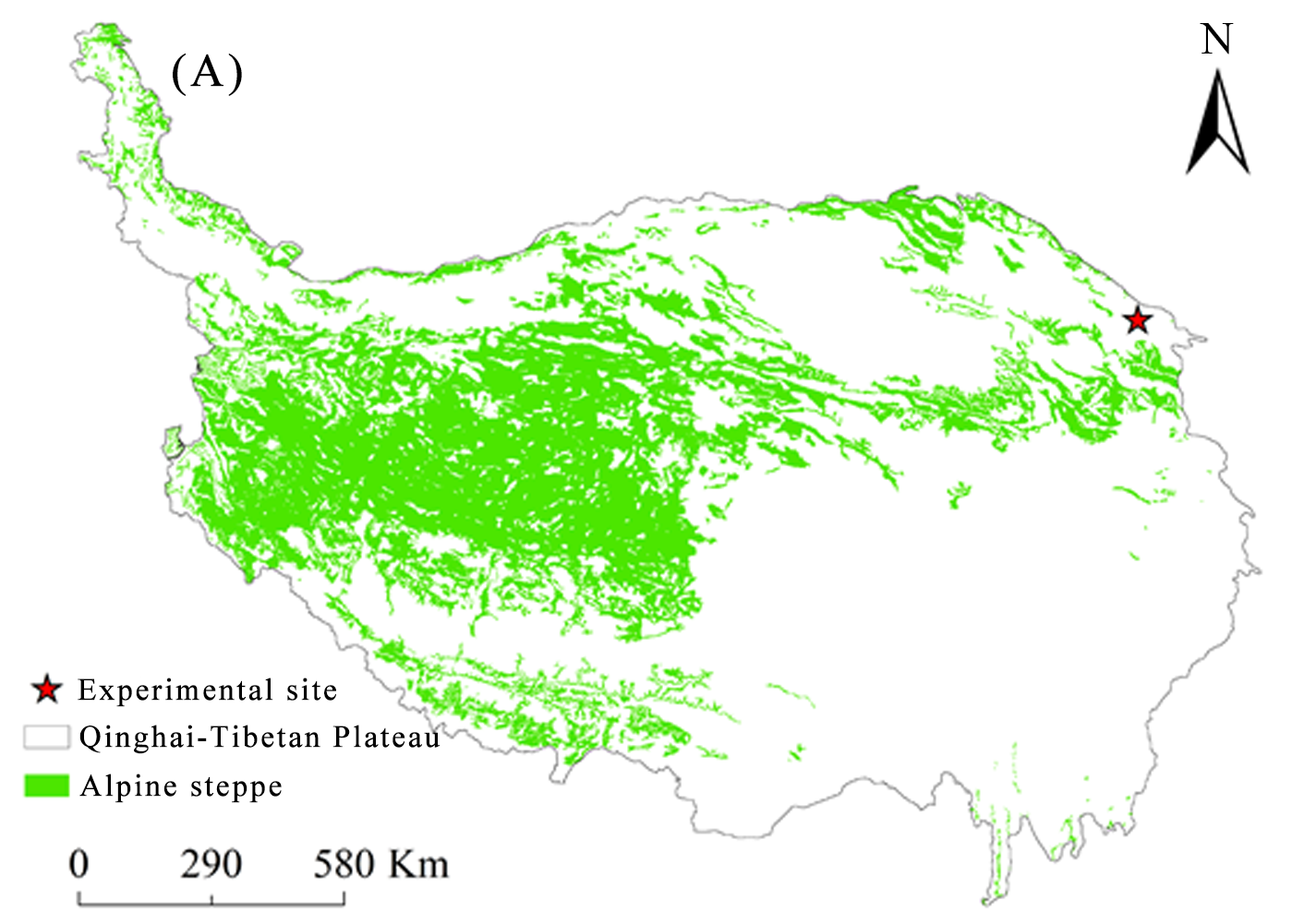


**Supplementary Figure 2.** Geographical location of the sampling sites in the present study.


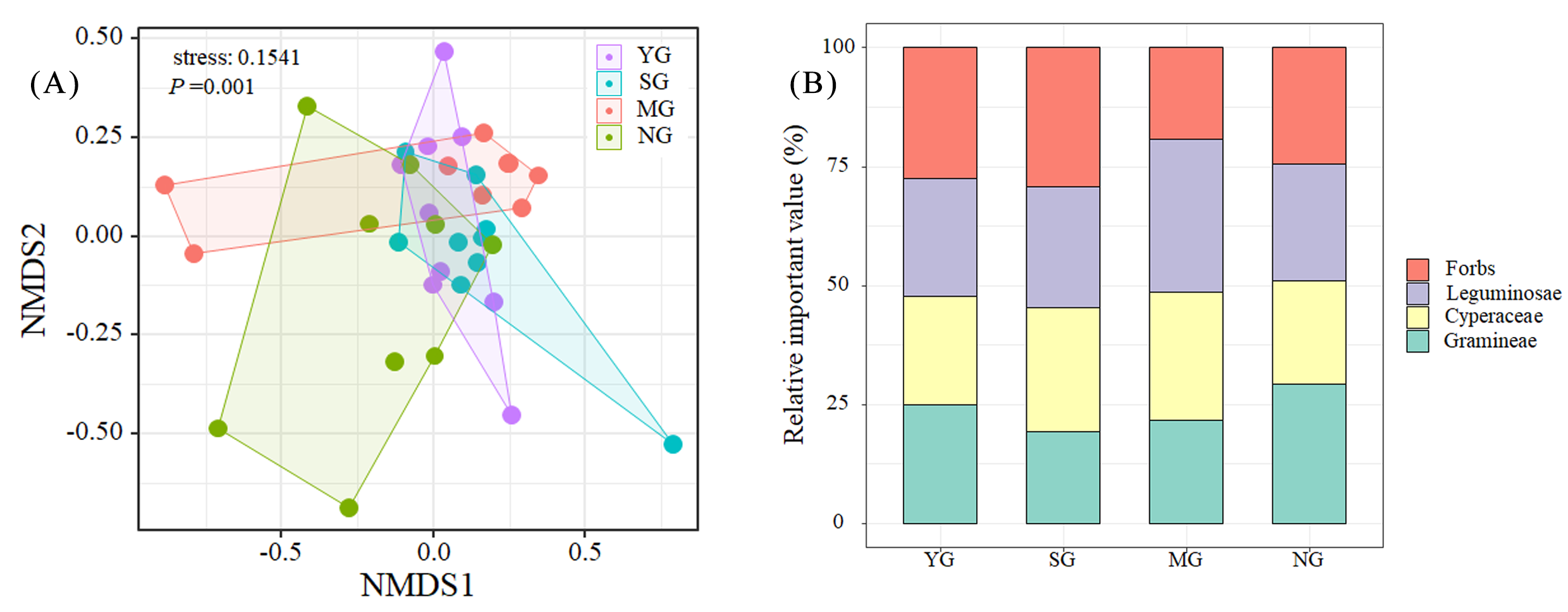


**Supplementary Figure 3.** Non-metric multidimensional scaling (NMDS) ordination of all sampling units indicating the relative differences in plants (A); Relative important values of plants under different herbivore assemblages (B). YG: Yak grazing; SG: Tibetan Sheep grazing; MG: Yak and Tibetan sheep mixed grazing; NG: No grazing.


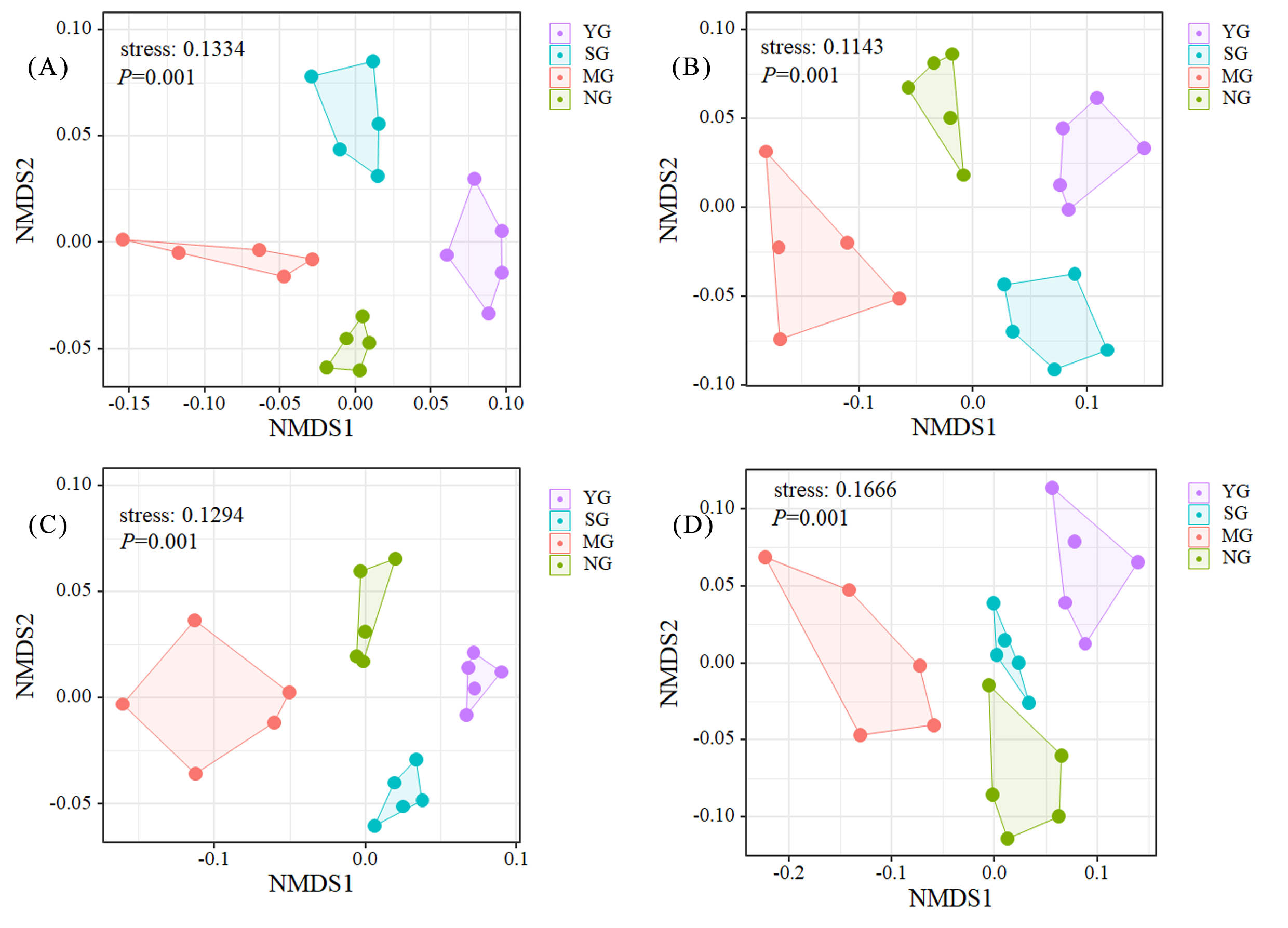


**Supplementary Figure 4.** Non-metric multidimensional scaling (NMDS) ordination of all sampling units indicating the relative differences in bacterial phyla Actinobacteria (A), Proteobacteria (B), Acidobacteria (C) and Planctomycetes (D) community compositions. YG: Yak grazing; SG: Tibetan Sheep grazing; MG: Yak and Tibetan sheep mixed grazing; NG: No grazing.


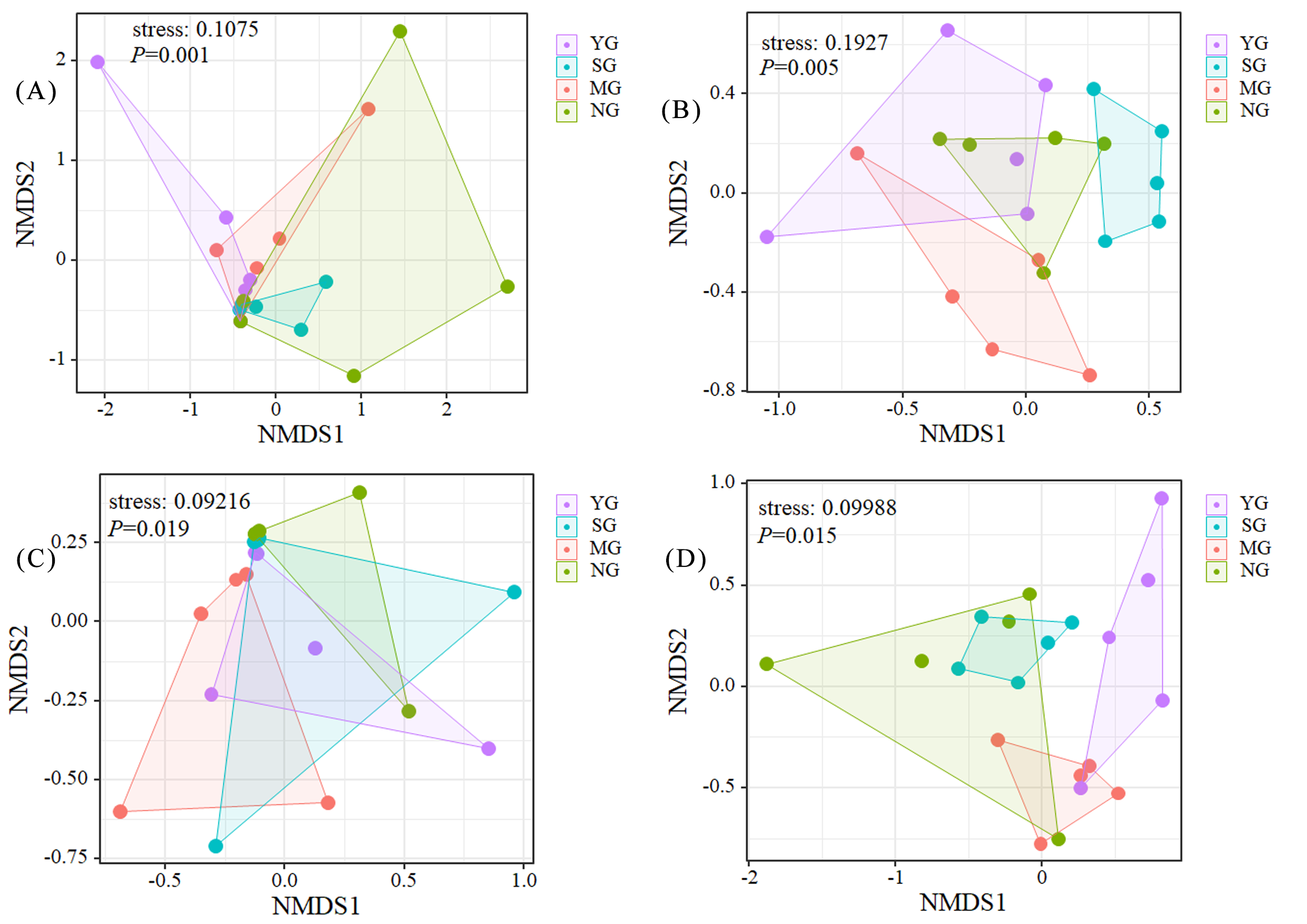


**Supplementary Figure 5.** Non-metric multidimensional scaling (NMDS) ordination of all sampling units indicating the relative differences in fungal families Clavicipitaceae (A), Nectriaceae (B), Pseudeurotiaceae (C) and Saccharomycetaceae (D) community compositions. YG: Yak grazing; SG: Tibetan Sheep grazing; MG: Yak and Tibetan sheep mixed grazing; NG: No grazing.


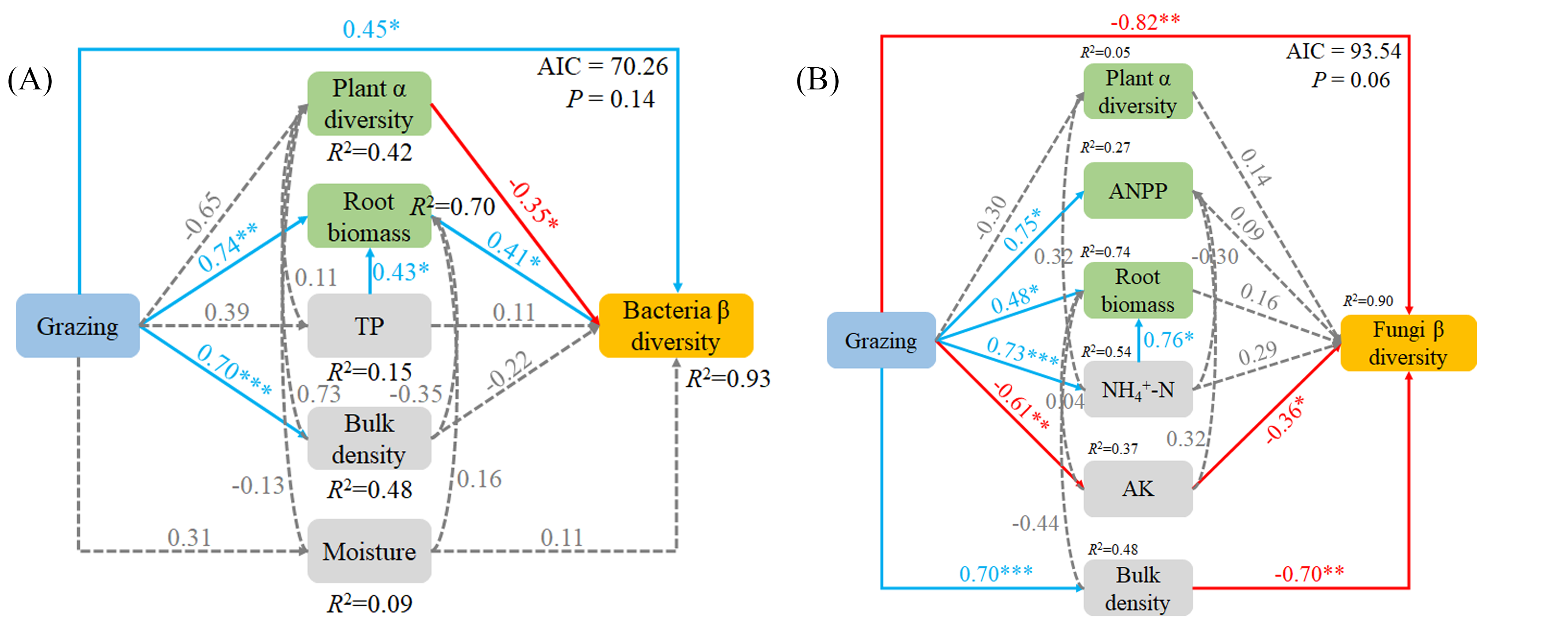


**Supplementary Figure 6.** Hypothetical causal model for structural equation modelling (SEM).

## Supplementary Tables

**Supplementary Table 1.** Relationships between soil microbial community and biotic and abiotic variables. Bold values represent significant relationships. TC: total soil carbon; TN: total soil nitrogen; C/N: carbon/nitrogen; TP: total soil phosphorus; NO3--N: soil nitrate; NH4+-N: soil ammonium; AP: soil available phosphorus; AK: soil available potassium; SBD: soil bulk density; ANPP: aboveground net primary productivity.

| Variables | Bacteria | | Fungi | |
| --- | --- | --- | --- | --- |
|  | *r*^2^ | *p* | *r*^2^ | *p* |
| TP (g·kg^-1^) | **0.356** | **0.022** | **0.388** | **0.012** |
| NO_3_^-^-N (mg·kg^-1^) | 0.171 | 0.210 | **0.357** | **0.025** |
| NH_4_^+^-N (mg·kg^-1^) | 0.165 | 0.227 | **0.530** | **0.005** |
| AP (mg·kg^-1^) | **0.661** | **0.001** | **/** | **/** |
| AK (mg·kg^-1^) | 0.076 | 0.522 | **0.360** | **0.037** |
| pH | 0.087 | 0.483 | 0.052 | 0.627 |
| Moisture (%) | **0.324** | **0.038** | 0.094 | 0.404 |
| SBD (g·cm^-3^) | **0.676** | **0.001** | **0.366** | **0.021** |
| Shoot biomass (g·m^-2^) | 0.012 | 0.916 | 0.110 | 0.333 |
| Root biomass (kg·m^-2^) | **0.347** | **0.032** | **0.322** | **0.038** |
| ANPP (g·m^-2^) | 0.088 | 0.442 | **0.301** | **0.046** |
| Plant α diversity | **0.516** | **0.003** | **0.306** | **0.047** |

# Supplementary Material

**Appendix S1.** Details of DNA extraction and high‐throughput sequencing methodology.

Soil DNA was extracted from 0.25 g using the HiPure Soil DNA Kit (Magen, Guangzhou, China) according to the manufacturer’s protocol. Twenty DNA extracts were obtained and then stored at -80°C for further analyses. DNA degradation and impurity were detected by 1% agarose gel electrophoresis, DNA purity was assessed by NanoDrop 2000 UV-vis Spectrophotometer (Thermo Fisher Scientific, Wilmington, DE, USA), and DNA concentration was determined on a Qubit 3.0 Flurometer (Thermo Fisher Scientific). The hypervariable V3-V4 region of the bacterial 16S rRNA gene was amplified with primer pairs 341F (5’-CCTACGGGNGGCWGCAG-3’) and 806R (5’-GGACTACHVGGGTWTCTAAT-3’) by an ABI GeneAmp® 9700 PCR thermocycler (ABI, Foster City, CA, USA). The fungal ITS1 region was amplified using the primers ITS1_F_KYO2 (50-TAGAGGAAGTAAAAGTCGTAA-30) and ITS86R (50-TTCAAAGATTCGATGATTCAC-30). HiSeq sequencing and PE250 sequencing strategy were used.
